# Editorial: Women in breast cancer: 2021

**DOI:** 10.3389/fonc.2023.1115949

**Published:** 2023-02-23

**Authors:** Elena Provenzano, Angela Toss

**Affiliations:** ^1^ Department of Histopathology, Addenbrookes Hospital and Cambridge Biomedical Research Centre (NIHR), Cambridge, United Kingdom; ^2^ Department of Oncology and Hematology, Azienda Ospedaliero-Universitaria di Modena, Modena, Italy; ^3^ Department of Medical and Surgical Sciences, University of Modena and Reggio Emilia, Modena, Italy

**Keywords:** breast cancer, original research, literature reviews, women, editorial, treatment

## Introduction

Welcome to the first Women in Breast Cancer special edition of Frontiers in Oncology. All the articles in the edition have a woman as lead and/or last author, highlighting the tremendous contribution female doctors and scientists are making to advance our understanding of breast cancer. The articles are summarized below and include original research, literature reviews and expert opinion on a breadth of topics including pre-clinical scientific research, prevention, pathological characterization of breast cancer and biomarkers, and surgical and oncological management.

## Preclinical research

Abraham et al. explored the role of Rho GTPases in invasion and metastasis by developing RhoC knockout breast cancer cell lines. The RhoC knockout cells showed increased cell-cell adhesion and barrier integrity with decreased expression of type 1 interferon-stimulated genes and a dampened response to interferon-alpha stimulation. RhoC GTPase is proposed as a potential therapeutic target in breast cancer.

Benczeet al. examined the effect of systemic desensitization of capsaicin-sensitive afferents in a triple negative mouse model of breast cancer and found an increase in early phase tumour growth accompanied by increased intratumoural vascular leakage in desensitized mice that subsided in later phases, supporting an anti-tumoural and vasoregulatory role for capsaicin-sensitive sensory nerves.

Correa et al. used miRNome profiling of breast cancer cell lines and identified 110 miRNAs common to all breast cancer subtypes. MYB and EZH2 were identified as potential targets for the breast cancer miRNome and this was confirmed in samples from breast cancer patients and in the TCGA dataset. Seven novel miRNAs were identified that act as regulators of MYB and EZH2 and have potential importance in breast cancer biology.

Harris et al. employed several advanced *in vitro*, ex vivo, and *in vivo* models, as well as over 50 human patient samples, to uncover a previously unstudied phenomenon by which platinum chemotherapy causes systemic VEGFR3-dependent lymphangiogenesis in both cancerous and healthy tissues. Their findings suggest that this lymphangiogenesis can enhance cancer cell invasion and metastasis. Additionally, they also showed that these effects can be mitigated with anti-VEGFR3 therapy, which may impact on-going efforts to clinically implement VEGFR3 inhibition.

Kraus et al. report on a novel therapeutic strategy, Sarah nanoparticles, that are multicore iron-oxide based nanoparticles that work by selective delivery of thermal energy to tumour cells following exposure to an alternating magnetic field. Therapeutic efficacy, toxicity and survival were evaluated in a 4T1 mouse model of breast cancer metastasis. The nanoparticles were found to accumulate in lung metastases and did not cause any toxicity. Treated mice had fewer lung metastases of smaller size than control mice, with a significant increase in survival. This proof-of-concept study provides a basis for development of this technology as a therapeutic approach for breast cancer.

Mahmoud et al. explored the expression pattern and the diagnostic value of the long intergenic ncRNA00511 (LINC00511) and its downstream microRNA (miR-185-3p) and the pathogenic significance of the onco-miR-301a-3p in naïve BC patients. Their analyses showed that lymph-node metastasis and advanced tumor grade were directly correlated with LINC00511, and that both LINC00511 and miR-301a-3p were positively correlated with the aggressiveness of BC.

## Prevention (primary and tertiary)

Ou et al. comprehensively analyzed the health losses caused by breast cancer attributable to secondhand smoke worldwide and estimated their trends with the updated Global Burden of Disease data. The results released that secondhand smoke remains a challenge to the patients’ longevity and quality of life.

Rujchanarong et al. focused their work on understanding if combined N-glycan biosignatures in breast stroma can further help to understand normal breast tissue at risk between black women and white women. Their study lays the foundation for understanding the complexities linking socioeconomic stresses and molecular factors to their role in ancestry dependent breast cancer risk and aggressiveness in black women.

Sim et al. presented the first known retrospective study on the effect of statin use and breast cancer recurrence in an Asian population. Similar to previous international studies, statin use was associated with a risk reduction in breast cancer recurrence, especially in patients who have ER+ and HER2- invasive breast cancer.

## Pathological and molecular characterization and biomarkers

Andrikopoulou et al. performed next generation sequencing to examine the mutational profile of breast cancer in women under 40 compared with older patients. PIK3CA and TP53 were the most common mutations in both groups of patients. CHEK2 and BRCA1 were the most common germline mutations identified and these were more frequently identified in younger women. No statistically significant differences were detected, however the sample size was small.

Dai et al. described the first case in literature of breast metastasis arising from rectal cancer pathologically diagnosed as a signet ring cell carcinoma and review the current literature on this rare event.

Hao et al. compared a quantitative dot-blot (QDB) method with routine immunohistochemistry for quantification of Ki67 to distinguish between luminal A and B ER-positive breast cancers. QDB is an ELISA-based test that can be performed on formalin-fixed paraffin embedded tissue. The QDB assay subtyped luminal breast cancers more effectively than Ki67 scoring by pathologists, and showed a better correlation with overall survival on multivariate analysis. The QDB-based assay provides objective and reproducible assessment of Ki67 levels and may offer an alternative to immunohistochemistry particularly in settings where gene expression-based prognostic tests for luminal breast cancer are unavailable.

Li et al. characterized the biological features of the main Triple Negative Breast Cancer histologic subtypes, comparing the clinicopathologic and genetic differences between invasive ductal carcinoma of no special type and special morphologic patterns.

Liu et al. developed a nomogram based on three daily available clinical parameters (lymphovascular invasion, axillary lymph node cortex thickness, and obliterated axillary lymph node fatty hilum), with good accuracy and clinical utility, to predict axillary lymph node metastasis.

Pernas et al. presented the findings of the AGATA study, a multi-institutional molecular screening programme in Spain (15). DNA sequencing of 74 cancer related genes was performed on FFPE samples from patients with advanced breast cancer. Somatic mutations were identified in 62.7% with recommendation for targeted therapy in 45%, although only 11% actually received the drug. The study demonstrated the feasibility of genomic testing of advanced breast cancer in a real-world setting.

Prasad et al. comprehensively profiled a large series of gynaecomastia samples for putative mammary diagnostic, predictive and prognostic markers. They found that hormone receptors, including oestrogen as well as androgen receptors were abundantly expressed within the intraductal luminal hyperplastic epithelium in gynaecomastia supporting the hormonal role in the pathogenesis and treatment. The authors concluded that Ki67 and cytokeratins can help in the differential diagnosis from histological mimics in the routine diagnostic work up.

Radziuviene et al. used hexagonal tiling analytics of digital image analysis to examine intratumoural heterogeneity and spatial aspects of the tumour microenvironment in HER2 borderline breast cancer and identified novel prognostic models based on computational intratumoural heterogeneity and immunogradient indicators of immunohistochemical biomarkers including ER, HER2 and CD8.

Saponaro et al. examined the association of NOD-like receptor protein 3 inflammasome markers and survival outcomes in invasive breast cancer and identified an association between increased expression of NLRP3 and TLR4 and worse disease-free survival. TLR4 overexpression was an independent prognostic factor for survival on multivariate analysis.

Truffi et al. looked at preoperative systemic inflammatory biomarkers and their potential role in predicting recurrence with a focus on ER-positive/HER2-negative breast cancer. They found low neutrophil-to-lymphocyte ratio, high lymphocyte-to-monocyte ratio and low platelet-to-lymphocyte ratio were all associated with improved survival outcomes. In ER-positive/HER2-negative breast cancer high platelet-to-lymphocyte ratio predicted worse outcome whereas high lymphocyte-to-monocyte ratio predicted better outcome. Inflammatory blood biomarkers are cheap and readily accessible across all health care settings and their potential value in risk stratification warrants further investigation.

Wang et al. retrieved data from the Surveillance, Epidemiology, and End-Results (SEER) database (2004-2015) to explore prognostic effect of clinicopathological features and treatment modalities on survival outcomes of primary breast signet ring cell carcinoma and mucinous breast adenocarcinoma patients. They found that primary breast signet ring cell carcinoma has unique clinical characteristics and poor prognosis compared with the mucinous breast adenocarcinoma group.

## Surgery

Blundo et al. looked at the safety of primary breast conserving surgery with delayed radiotherapy in a cohort of women presenting with breast cancer during the first trimester of pregnancy (21). Pregnancy outcomes were good and there were no perioperative complications. None of the patients developed ipsilateral local recurrence in the first 5 years after diagnosis. Four women developed ipsilateral breast cancer 6 or more years after the primary diagnosis; this was not significantly different to a control group treated with primary mastectomy. Primary breast conserving surgery was deemed to be a safe option for women diagnosed with breast cancer in early pregnancy.

Zhao et al. developed a nomogram to enable the estimation of the preoperative risk of positive breast-conserving surgery margins. The nomogram they propose provides a valuable tool for identifying high-risk patients who might have to undergo a wider excision.

Zhao et al. provided extensive real-world evidence on breast surgery in China. Using data from a survey in 2015, they found that breast cancer occurs at a younger age in Chinese women, the primary type of surgical procedures was mastectomy in 2015 and there was a wide surgery cost range both across and within geographic regions.

## Clinical management

Carter et al. explored the literature on the role of oncolytic viruses in treatment of breast cancer. At present, fourteen oncolytic viruses have been investigated in 18 published clinical trials, however these trials included a small number of patients, only some of whom had advanced breast cancer. Future breast cancer specific trials are required to determine the utility of this novel approach.

George et al. present a comprehensive review of the currently available Cyclin Dependent Kinase 4 and 6 inhibitors palbociclib, ribociclib and abemaciclib. Differences in their substrate selectivity and pharmacodynamics, side effect profiles, resistance mechanisms and central nervous system penetrability are described that need to be considered when initiating therapy.

Gwark et al. evaluated the effectiveness of neoadjuvant chemotherapy for avoiding axillary lymph node dissection, compare with neoadjuvant endocrine therapy in premenopausal patients with ER-positive/HER2-negative, lymph node-positive breast cancer. Neoadjuvant chemotherapy led to a significantly lower axillary lymph node dissection rate and mean number of removed and positive lymph nodes than those obtained through neoadjuvant endocrine therapy. Moreover, the axillary pathologic complete response (pCR) rate was significantly increased.

Pinilla et al. presented an overview of contemporary practice, and promising future trends in the management of early TNBC. The review presents the growing evidence for less-developed agents and predictive biomarkers and proposes a framework for the personalized management of TNBC based upon the integration of clinico-pathological and molecular features to ensure that long term outcomes are optimized.

Schettini et al. conducted a window of opportunity study looking at the effect of a short course of olaparib in patients prior to receiving standard neoadjuvant chemotherapy for locally advanced triple-negative breast cancer called the OLTRE trial, specifically comparing subgroups of g-BRCA-wild type triple negative cancer against g-BRCA-mutant HER2 negative cancer. The study identified partial clinical and radiometabolic responses in both g-BRCA-mutant and g-BRCA-wild type populations suggesting olaparib has a role in the treatment of early-stage triple negative breast cancer independent of gBRCA status.

Stucci et al. reviewed the literature on neoadjuvant and adjuvant therapy for HER2-positive breast cancer including findings of the key clinical trials. They specifically address the situation of regulatory approval in Italy for anti-HER2 therapy in the neoadjuvant and adjuvant settings, and suggest an algorithm to aid decision making based on tumour stage and nodal involvement.

Tolaney et al. conducted a phase 1b study evaluating the safety and tolerability of Abemaciclib in combination with endocrine therapy for patients with hormone-receptor positive, HER2-negative metastatic breast cancer and concluded that Abemaciclib given with endocrine therapy exhibited a manageable safety profile and promising anti-tumour activity in this population of patients.

## Conclusion

The number and quality of submissions received, across the whole spectrum of topics, from basic science to prevention to treatment of early and metastatic disease, showcase how women are leading the fight against breast cancer. A second edition of women in Breast Cancer is already in progress. We hope you find the articles interesting and educational.

## Author contributions

All authors listed have made a substantial, direct, and intellectual contribution to the work and approved it for publication.

